# Notes and News

**Published:** 1894-03-10

**Authors:** 


### Notes and News.

Collected and Communicated.

### MEETINGS OF THE WEEK.

Royal Sea Bathing Infirmary: Annul meeting, 3 p.m., March 12th.

Home Hospitals Association: Annual Meeting March 15th, at 16, Fitzroy Square, at 4 p.m.

The Dental Hospital of London: Annual meeting, 5.30 p.m., March 15th.

The close of the year at Montreal was marked by an important event. This was the opening of the Royal Victoria Hospital, which was presided over by his Excellency the Viceroy. As long ago as 1887, in commemoration of the Queen's Jubilee, Lord Mount-stephen and the Hon. Sir Donald Smith provided the munificent sum of one million dollars for the establishment of the hospital. The building was commenced in 1891 on the margin of the park, on a suitable elevation No pains or money have been spared to equip the hospital in the most efficient manner. An excellent staff of physicians have granted their services, and externally and internally the Royal Victoria Hospital does credit to the city and to the generous founders and supporters.

We should like to know what is to be done to carry into effect the recommendation of General Buller's Committee of Inquiry respecting the insanitary state of the North Camp Hospital at Aldershot. This hospital consists of a lot of old Crimean huts built forty years ago, and totally unfit, even then, for the accommodation of the sick according to modern ideas. The *Globe* of December 23rd called attention to the fact that, notwithstanding the grant of money for the purpose of reconstructing the hospital, and notwithstanding the continuance of typhoid and other diseases attendant on insanitation in that part of the camp, both soldiers and medical officers continue to be housed there. While these complaints continue to be gathering, plenty of money has been spent on new churches, gymnasia, and engineers' barracks.

The Chelsea Hospital for Women will now be wearing a new dress, for nothing short of a very perfect garment will pass the test of the search light of inquiry which is being brought to bear upon it. The Chelsea Vestry, the Local Government Board, the Home Secretary, and the House of Commons are all to be asked to help in the reformation.

We are glad to learn that the agitation against the management of the Aberdeen City Hospital has been laid at rest. Whilst all hospitals do well to court public opinion, it is to be regretted that irresponsible criticism cannot be kept out of the public press, as by it much time is wasted and much harm done.

Much encouragement is given and excellent facilities are afforded to English visitors to the International Medical Congress at Rome. Reduced railway rates are charged, both to medical men and those accompanying them. The Secretary for the English Committee (Mr. G. Makins, 47, Charles Street, Berkeley Square) issues passes to medical men and their relatives.

A prize of £1,000 is offered by Count Orloff-Davidoff for a certain cure of the plague amongst horned cattle. The Judging Committee sits at St. Petersburgh. The competition is open to the whole world, and the prize is to be awarded on Janaury 1st, 1899. It will not be surprising if this large prize remains unawarded. Discoverers are seldom inspired by the prospect of gain, and discoveries are nearly as often accidentally made as not.

A rather absurd piece of red-tapeism has been experienced by the superintendent of a lunatic asylum in the United States. Dr. Blumer, the gentleman in question, has been at his post for seven years. Two years after his tenure of office had commenced the post he held was placed on the competitive schedule. After the lapse of a further five years Dr. Blumer is now called to present himself for examination as to his fitness to hold the position to which he was elected.

By the generosity of M. G. Solvay, two important Institutes have been established at Brussels, namely, a University Institute of Physiology and an institute especially designed for carrying on electro-biological researches. In May last, M. Solvay presented the town with a sum of 200,000 francs, for the erection and equipment of the University building, stipulating only that the University should provide courses in physiological chemistry, and medical physics relating to the connection between physiology and electricity. This condition was laid down with the object of improving the instruction at the University, and developing the spirit of investigation in the minds of students, and giving them the ability to carry on physiological researches independently in a special laboratory. For students thus trained, and desiring to apply their knowledge to research of a special kind, the Solvay Institute has been established. At the inauguration of this institution, on December 14th, M. Solvay delivered an address on the *role* of electricity in the phenomena of life. He remarked that his conviction was that the phenomena of real life could probably be explained by the act of physical force, and among these forces electricity played an important part. It was to obtain evidence on this point, by the observation and study of facts, that M. Solvay was led to found the institution that bears his name.

